# Endothelial S1pr2 regulates post-ischemic angiogenesis via AKT/eNOS signaling pathway

**DOI:** 10.7150/thno.71585

**Published:** 2022-07-04

**Authors:** Caixia Zhou, Yashu Kuang, Qinyu Li, Yunhao Duan, Xiuxiang Liu, Jinnan Yue, Xiaoli Chen, Jie Liu, Yuzhen Zhang, Lin Zhang

**Affiliations:** 1 Key Laboratory of Arrhythmias of the Ministry of Education of China, Research Center for Translational Medicine, Shanghai East Hospital, Tongji University School of Medicine, Shanghai, 200120, China.; 2 Postgraduate training base in Shanghai Gongli Hospital, Ningxia Medical University, Ningxia, 750004, China.

**Keywords:** Sphingosine 1-phosphate receptor 2, Endothelial cells, Hindlimb ischemia, Angiogenesis

## Abstract

**Aims:** It is important to understand the mechanism that regulates post-ischemic angiogenesis and to explore a new therapeutic target for an effective improvement of revascularization in peripheral artery disease (PAD) patients. Post-ischemic angiogenesis is a highly orchestrated process, which involves vascular endothelial cells (ECs) proliferation, migration and assembly into capillaries. We found a significant reduction of S1pr2 (sphingosine 1-phosphate receptor 2) in endothelial cells after hindlimb ischemia (HLI). We thus hypothesized that EC-S1pr2 might be involved in the regulation of post-ischemic angiogenesis and blood flow recovery during peripheral arterial disease (PAD).

**Methods and Results:** We generated both EC-specific S1pr2 loss-of-function and S1pr2 gain-of-function mice. Our study showed that EC-specific S1pr2 loss-of-function significantly enhanced post-ischemic angiogenesis and improved blood flow recovery upon femoral artery ligation, whereas the EC-specific S1pr2 gain-of-function severely hindered post-ischemic angiogenesis and reduced blood flow recovery in ischemic limbs. We next identified that S1pr2 inhibited AKT/eNOS signaling pathway, and thus inhibited EC proliferation/migration and angiogenic activity. As expected, pharmacological inhibition of S1pr2 by JTE013 improved post-ischemic angiogenesis and improved blood flow perfusion after femoral artery ligation. Moreover, we developed RGD-peptide magnetic nanoparticles packaging S1pr2-siRNA which specifically targeted ECs and achieved an efficient silencing of S1pr2 expression in ECs *in vivo*. This EC-targeted strategy to dampen S1pr2 significantly enhanced post-ischemic angiogenesis and boosted blood perfusion after HLI, supplying a novel therapy target for patients with peripheral arterial disease.

**Conclusions:** This present study demonstrates that EC-expressing S1pr2 tightly controls post-ischemic angiogenesis and blood flow perfusion recovery. This research provides a novel strategy for EC-target knockdown of S1pr2 as a new therapeutic intervention for patients with peripheral artery disease.

## Introduction

Peripheral arterial disease (PAD) in the legs or lower extremities is a common circulatory problem in which a chronic narrowing of arteries results in the reduction or blockage of blood flow to the limb, leading to a loss of functionality. PAD affects around 200 million people worldwide, and its occurrence rate is sharply rising in the elderly population 1. Although PAD is associated with high morbidity and mortality rates especially in elderly people aged 64 years or over, the clinic efficacy of PAD therapy is unsatisfactory 1. Therefore, there is an urgent need to develop novel strategies and therapy targets for PAD. Post-ischemic angiogenesis and revascularization are key reparative processes upon arterial narrowing or occlusion 2, 3. A better understanding of the mechanism that regulates post-ischemic angiogenesis might help us to develop a novel therapeutic target for an effective revascularization in PAD.

Angiogenesis is a multistep process that involves migration, proliferation and lumen formation of endothelial cells (ECs) to form nascent blood vessels from the existing vasculature under both physiological and pathophysiological conditions 4. In response to tissue ischemia, ischemic tissues release angiogenic factors and boost angiogenesis to generate new vessels 5. During angiogenesis, nascent vessels recruit mural cells including smooth muscle cells, pericytes and fibroblasts, to form functional microvasculature, which improves blood flow perfusion in ischemic tissue 5. Among angiogenic factors, S1P is a key regulator of angiogenesis via its receptors. It has been shown that sphingosine 1-phosphate receptor 1 (S1pr1) tightly controlled endothelial integrity and developmental angiogenesis 6, 7. Besides the expression of S1pr1 in ECs, other studies revealed that sphingosine 1-phosphate receptor 2 (S1pr2) was expressed in vascular endothelial cells as well 8. Previous *in vitro* studies revealed that S1P/S1pr2 signaling was involved in EC dysfunctions 9, 10, suggesting that S1pr2 might play a role in post-ischemic angiogenesis. However, the cell-specific effect of S1pr2 on post-ischemic angiogenesis and revascularization in peripheral arterial disease is not well known. In this study, we sought to investigate whether and by which mechanism S1pr2 controlled post-ischemic angiogenesis and influenced blood flow recovery during PAD.

Here, we reported that EC-S1pr2 deletion led to an improvement in post-ischemic angiogenesis and an enhancement in blood flow recovery in ischemic tissues, and* vice versa*. Pharmacological blockade of S1pr2 signaling by JTE013 or RGD-peptide magnetic nanoparticles packaging S1pr2-siRNA to specifically target ECs significantly improved post-ischemic angiogenesis and enhanced blood flow perfusion in ischemic tissues. This study provides a future promising EC-target therapy for peripheral arterial disease through the S1pr2 signal pathway.

## Methods

### Generation of endothelial cell-specific S1pr2 loss-of-function or gain-of-function transgenic mice

The conditional *S1pr2* gain-of-function (*S1pr2^Tg/+^*) mouse was generated by inserting a *S1pr2*-3Xflag-IRES-EGFP cassette into the *Rosa26* locus. The conditional *S1pr2* knock-out (*S1pr2^flox/flox^*) mouse was generated by inserting loxP sites flanking exon 2 in *S1pr2* genomic DNA. The conditional *S1pr2* knock-out (*S1pr2^flox/flox^*) mice or *S1pr2* gain-of-function (*S1pr2^Tg/+^*) mice were crossed with tamoxifen-inducible *Cdh5* promoter-driven Cre line (*Cdh5-Cre^ERT2^*) 11 to generate endothelial-specific *S1pr2* knock-out mice (*Cdh5-Cre^ERT2^*;*S1pr2^flox/flox^*) or *S1pr2* gain-of-function mice (*Cdh5-Cre^ERT2^*;*S1pr2^Tg/+^*), respectively. 8-week-old male *Cdh5-Cre^ERT2^*;*S1pr2^wt/wt^* or* Cdh5-Cre^ERT2^*;*S1pr2^+/+^* littermates with C57BL/6J background were applied in all experiments as *WT* control mice for *Cdh5-Cre^ERT2^*;*S1pr2^flox/flox^* or* Cdh5-Cre^ERT2^*;*S1pr2^Tg/+^*, respectively. Tamoxifen was administrated via intra-peritoneal (100 mg/kg, i.p. every other day for 4 times) injection one week before the operation. All animals were randomized into different groups and analyzed in a blinded fashion to avoid sampling bias. All animal procedures were operated in accordance with the Institutional Animal Care and Use of Laboratory Animals approved by the Tongji University Institutional Animal Care and Use Committee with license number TJLAC-0126-026.

### Induction of hind limb ischemia animal models

Hind limb ischemia (HLI) was induced in 8-week-old mice as previously reported 12. Briefly, mice were anesthetized with an injection of 1% pentobarbital sodium (40 mg/kg, i.p.). Depilatory cream was used to shave off the hair of the limbs and the operation area was sterilized with 70% ethanol. A vertical skin incision was made over the femoral artery from the inguinal ligament to the popliteal bifurcation. The femoral artery on the left side was isolated and doubly ligated using two discontinuous 6-0 silk sutures. Limbs in sham mice were opened, dissected, and closed without vessel ligature and excision. The femoral artery on the right side was sham-operated as a control for HLI. In pharmacological experiments, S1pr2 inhibitor, JTE013 (3.0 mg/kg/day, Cayman #10006440-5), and vehicle control, DMSO, were administrated by intra-peritoneal (i.p.) injection every day for 14 days after HLI.

### Perfusion imaging

A laser doppler imaging system (moor instruments, United States) was applied to measure blood flow perfusion before and 0, 7, 14 days after the femoral artery ligation. The calculated blood flow perfusion was expressed as a ratio of blood perfusion in the ischemic limb to the sham operation limb measured by moorLDI laser doppler imager review V 6.0 analysis software.

### Functional Scoring

Function and tissue damage scoring was performed using the Tarlov score, ischemia score and ambulatory impairment score, as previously reported (Table 1-3) 13.

### Histology

The ischemic hindlimb muscle tissues were collected 14 days after HLI. The muscles were fixed, dehydrated, embedded, and sectioned into 6 μm-thick pieces. The tissue sections were stained with hematoxylin&eosin (H&E) and Sirius Red. Immunostaining was performed on cryostat 8 μm-thick sections by using various antibodies, including anti-smooth muscle actin antibody (Proteintech, #00098013), biotinylated-isolectin B4 antibody (IB4, Vector Laboratories, B-1205), and their corresponding secondary antibodies. Nuclei were stained with DAPI (Sigma, #D9542). To evaluate capillary vessel density, the relative capillary density by calculation of the isolectin B4 positive capillary numbers per high-power microscopic field was calculated in IB4-staining muscle sections. L-NAME (Sigma Aldrich, #N5751), LY294002 (Selleck, #S1105), Anti-phospho-eNOS (Abcam, #ab184154) were commercially purchased from companies. Anti-Phospho-AKT (#4060), Anti-AKT (#9272), and Anti-eNOS (#32027) were purchased from Cell Signaling Technology. Anti-S1pr2 antibody (21180-1-AP) was purchased from Proteintech.

### Cell culture

Human umbilical vein endothelial cells (HUVECs, ATCC) were cultivated in EGM2 (Endothelial cell growth medium 2, PromoCell) and cells within the 8_th_ passage were used for *in vitro* experiments. HUVECs were infected with S1PR2 overexpressing lentivirus and its corresponding NC empty control lentivirus, and S1PR2 shRNA lentivirus and its corresponding scramble control lentivirus. The efficiency of S1PR2 knockdown and overexpression without alteration on S1PR1 mRNA levels in HUVECs was confirmed by quantitative real-time PCR analysis, as shown in **Figure S1A-D**. A mouse cardiac microvascular endothelial cell line (mCMVECs) was also used *in vitro* experiments, as previously reported 14.

Boyden chamber and scratch wound healing assay were applied to assess cell migration. Briefly, in transwell assays, ECs were placed on the upper layer of Boyden chamber (Falcon 353097) assay, and culture medium EGM2 containing VEGF (1 ng/μL) was placed below the cell-permeable membrane, with addition of 5-FU (1 μg/mL, Selleck) to inhibit cell proliferation. After 4-h incubation, the cells that have migrated through the membrane were stained by crystal violet solution and counted. In scratch wound healing assay, HUVECs were plated in plates to reach 90% confluence. A vertical wound scratch was generated and HUVECs were then cultured with FBS-reduced DMEM medium containing 5-FU (1 μg/mL, Selleck). At designated times, the wound of HUVEC culture was imaged and the wound closure rate was assessed.

For the measurement of NO, Griess reaction method was used to measure the production level of NO in culture medium. The NO production level was measured according to the instructions of the Nitric Oxide Assay Kit (Beyotime S0021S).

MTT assay was used for cell proliferation assay. Briefly, HUVECs were plated in flat-bottom 96-well cell culture plates for 48 h. 10 µl MTT (5 mg/mL) solution (Sigma) was then added to each well followed by a 4-h incubation at 37 ℃, and the media was replaced with a 100 µL mixture of DMSO. The cell culture plates were shaken for 10 min at room temperature and then monitored by a microplate reader (Bio-Rad) at 490 nm for the measurement of the absorbance in each well.

For tube formation assay, the matrigel with a volume of 50 µL was added to each well of a 96-well cell culture plate on ice and then was incubated at 37 °C for 0.5 h in order to allow matrigel to solidify. HUVECs were resuspended in the desired culture medium at 3 × 10^5^ cells/mL. 50 µL of cell suspension (1.5 × 10^4^ cells) were plated into each well onto the solidified matrigel gel. The assay plate was incubated at 37 °C, 5% CO_2_ for 4 to 8 h, and tube formation was observed by a phase-contrast light microscope. The total tube counts were measured using ImageJ software.

For the fibrin gel bead assay 15, HUVECs were mixed with Cytodex3 microbeads (Amersham Pharmacia Biotech) at a concentration of 400 cells/bead in EGM-2 (PromoCell) overnight. Cell-coated beads were washed and then cultured in a fibrin gel containing fibrinogen (Sigma, 2.5 mg/mL), aprotinin (Sigma, 0.15 units/mL), and thrombin (Sigma, 0.625 units/mL) and allowed to clot in 24-well tissue culture plates. Fibroblasts were seeded on the top of the fibrin gel as a feeder layer and the medium was refreshed every other day. The sprouting of HUVECS was observed by a phase-contrast light microscope. The number of sprouts, branched sprouts and scattered cells were counted for quantification by ImageJ software.

For isolation primary ECs, 8-week-old mice were sacrificed and their limb muscles were isolated aseptically and minced finely, and then digested in collagenase I (1 mg/mL, Sigma Aldrich, #C6885), and DNase I (60 U/mL, ThermoFisher #EN0521) at 37 °C at 100 rpm for 1 h. After passing through a 70-μm nylon strainer, the single-cell suspension of the gastrocnemius muscle was incubated in 0.1% bovine serum albumin/PBS with anti-CD31 antibody (BD Pharmingen 553370) which was conjugated with Dynabeads (Invitrogen, Carlsbad, CA), and rotated for 20 min. Primary endothelial cells were then separated in a magnetic separator and used for further quantitative analysis of gene expression by real-time PCR.

### RNA Extraction, RNA microarray, and Real-Time PCR for gene expression

Total RNA from hearts or ECs was extracted by Trizol (Invitrogen) following the manufacturer's instructions and used for RT-PCR (the primer list was supplied in Table 4). RNA quantity and quality were measured by NanoDrop ND-1000.

### Western blot

The total cell proteins of HUVECs were extracted and resolved by 10% SDS-PAGE gels. Proteins were electrophoretically transferred to PVDF membranes (Millipore, USA) for western blotting. The membranes were blocked in 5% skim milk for 2 h at room temperature and then incubated with the corresponding primary antibodies overnight at 4 ºC with rotation. The primary antibodies included Anti-Phospho-AKT (CST, #4060), Anti-AKT (CST, #9272), Anti-eNOS (CST, #32027) and phospho-eNOS (Abcam, #ab184154). After incubation with the corresponding peroxidase-conjugated secondary antibody, the signals were visualized using a chemiluminescence kit (Cell Signaling Technology).

### Controlled and targeted delivery of siRNA into endothelial cells via RGD-peptide magnetic nanoparticle *in vivo*

The EC target delivery of S1pr2-siRNA by RGD-peptide magnetic nanoparticles was constructed as previously reported 14, 16. The knockdown efficiency of siRNA against S1pr2 without alteration on S1pr1 mRNA levels was firstly confirmed in mCMVECs by quantitative real-time PCR analysis, as shown in **Figure S1E-F**, and then S1pr2-siRNA was packaged into RGD-nanoparticles. We constructed RGD-peptide containing magnetic nanoparticles consisting of two main parts, the Fe_3_O_4_ core part, and RGD-peptide. The core part magnetic Fe_3_O_4_ makes nanoparticles entering the hind limb ischemic area more efficiently by using a magnet on top of the hindlimbs. RGD-peptide worked as an integrin specificity ligand leading to the enrichment of the nanoparticles towards endothelial cells. Synthetic S1pr2-siRNA or scramble control (2 mg/kg each, Genomeditech) was mixed with nanoparticles at the ratio of 10 µg siRNA:50 μg nanoparticles in total 100 μL normal saline at room temperature for 1 h which allows siRNA to incorporate into the inner interspace of nanoparticles. The RGD-peptide nanoparticles were injected by the tail vein at day 1, 4, 7 and 10 following HLI. The blood flow perfusion was evaluated 7 and 14 days following HLI.

### Statistics

All continuous data were represented as means ± standard error of the mean (S.E.M.) for at least three independent assays unless otherwise noted. The student's t-test was used for comparisons between two groups. One-way or two-way ANOVA analysis followed by Tukey was applied in multiple comparison analyses. P < 0.05 was considered as statistical significance in this study. All data were checked for normality and equal variance before using parametric tests. All analyses were performed with SPSS 11.0 (SPSS. Inc) for Windows.

## Results

### S1pr2 is expressed in vascular endothelial cells and down-regulated upon HLI

**Figure 1:**
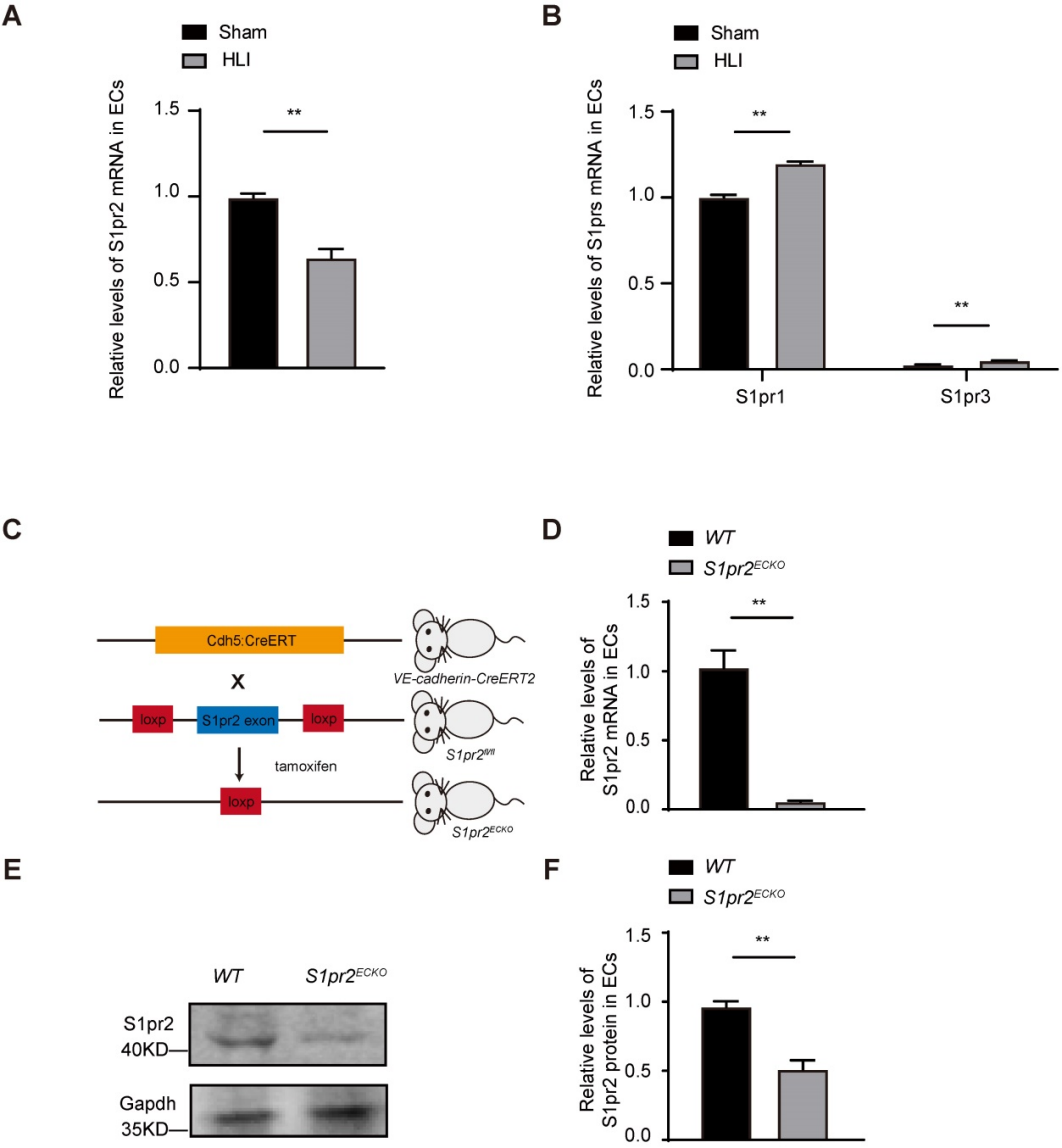
** S1pr2 is expressed in vascular endothelial cells and down-regulated after HLI. A,** S1pr2 expression in endothelial cells (ECs) of gastrocnemius muscles after HLI operation (n = 5). **B,** Relative mRNA expression levels of S1pr1 and S1pr3 in endothelial cells of gastrocnemius muscles obtained from mice after sham or HLI operation (n = 3). **C,** Generation of EC-specific S1pr2 loss-of-function mice.** D**, Relative mRNA expression levels of S1pr2 in ECs of *WT* and *S1pr2*^ECKO^ mice (n = 4). **E** and** F,** Western blotting analysis of S1pr2 protein levels in ECs from *S1pr2*^ECKO^ mice or *WT* control mice, with their quantification (n = 3). Data are mean ± SEM. n.s indicates not significant. *P < 0.05; **P < 0.01.

Angiogenesis and revascularization are the key restorative mechanisms of blood flow recovery and tissue repair after ischemia 2, 3. It has been shown that the expression of S1P receptors (S1prs) in vascular endothelial cells played an essential role in angiogenesis. However, the effect of EC-S1prs on post-ischemic blood flow recovery and tissue repair is not well known 8. In this study, we induced hindlimb ischemia (HLI) by ligation of the mouse femoral artery, which led to reduction of blood flow followed by post-ischemic angiogenesis and tissue repair. Our results showed that the S1pr2 expression in ECs significantly decreased in the ischemic hind limb, in comparison with the sham hindlimb, although other S1P receptors (including S1PR1, S1PR3) were moderately changed after HLI (**Figure 1A-B**). These results indicates that EC-S1pr2 might be involved in the regulation of blood perfusion recovery and tissue repair after hindlimb ischemia.

### Specific endothelial S1pr2 loss of function enhances post-ischemic angiogenesis and blood flow perfusion

**Figure 2:**
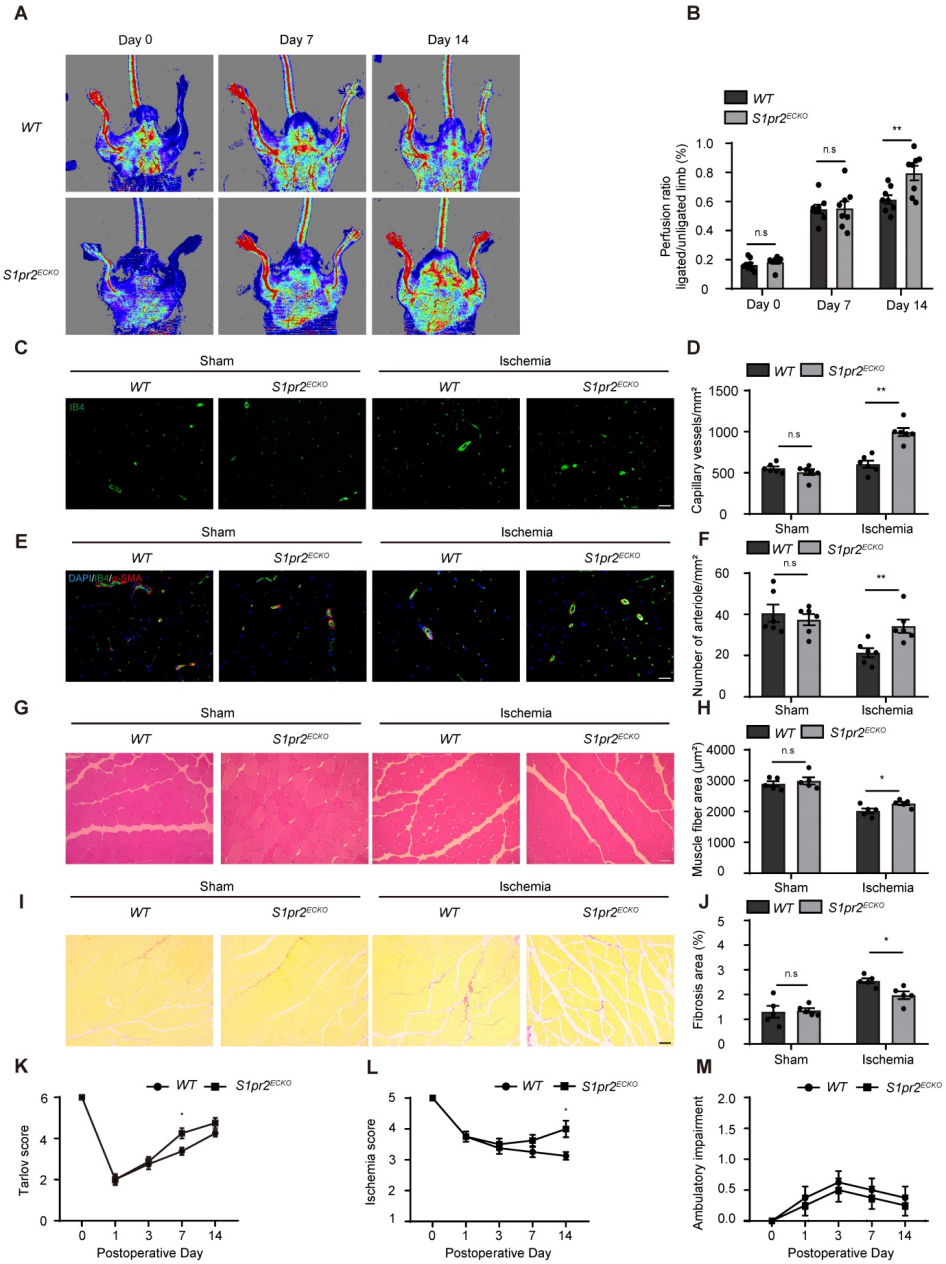
** Specific endothelial S1pr2 loss-of-function enhances post-ischemic blood flow perfusion and angiogenesis in hindlimbs. A**, Representative laser Doppler images show improved blood flow perfusion in *S1pr2*^ECKO^ mice compared with *WT* control mice. **B**, Cumulative results for *WT* mice (n = 8) and *S1pr2^ECKO^* mice (n = 8) are shown as the ratio of blood flow in the ischemic limb to that in the non-ischemic limb at each time point. **C-D**, Representative images of isolectin-B4 staining of gastrocnemius muscles in *WT* and *S1pr2^ECKO^* mice after sham or HLI operation **(C)**, with quantification of capillary density in gastrocnemius muscle after sham or HLI operation (D) (n = 6). **E-F**, Representative images of α-SMA staining of gastrocnemius muscles *WT* and *S1pr2^ECKO^* mice after sham or HLI operation **(E)**, with quantification of arteriole density in gastrocnemius muscle** (F)** (n = 6). **G-H**, Representative images of H&E staining of gastrocnemius muscles in *WT* and *S1pr2^ECK^*^O^ mice **(G)**, with quantification of muscle fiber area after sham or HLI operation (H) (n = 5). I-J, Representative images of Sirius red-stained of gastrocnemius muscles in *WT* and *S1pr2^ECKO^* mice (I), with quantification of the percentage of fibrotic tissue in gastrocnemius muscles (J) (n = 5). K-M, Functional assessment of ischemic muscle over follow-up. Cumulative results for *WT* mice and *S1pr2^ECKO^* mice are shown graphically as Tarlov score (K), ischemia score (L), and ambulatory impairment score (M) (n = 8). Scale Bars: C and E, 50 μm; G and I, 100 μm. Data are mean ± SEM. n.s indicates not significant. *P < 0.05; **P < 0.01.

To clarify whether EC-S1pr2 influences post-ischemic revascularization and tissue repair* in vivo,* we first generated EC-specific *S1pr2* knock-out mice (*S1pr2^ECKO^*) by intercrossing* the VE-cadherin-Cre^ERT2^* line with *S1pr2^flox/flox^* mice (**Figure 1C**). A sharp reduction in *S1pr2* mRNA and protein levels was observed in ECs of *S1pr2^ECKO^* mice after tamoxifen treatment, as shown by RT-qPCR and western-blotting (**Figure 1D-F**). Laser doppler imaging system showed that mean ratios of blood perfusion in ischemic hindlimbs to non-ischemic hindlimbs of the blood flow perfusion in ischemic hindlimbs of *S1pr2^ECKO^* mice was significantly higher than *WT* mice (**Figure 2A-B**), demonstrating that EC-S1pr2 might be involved in the regulation of post-ischemic angiogenesis and revascularization. We next explored the effect of EC-S1pr2 loss-of-function on post-ischemic angiogenesis. The capillary density was evaluated by immunostaining of isolectin B4 (IB4) in the gastrocnemius muscles at day 14 after HLI. Although loss of endothelial S1pr2 did not affect baseline muscle vascular density in hindlimbs, *S1pr2^ECKO^* mice significantly increased capillary vessel density in ischemic hindlimbs compared with* WT* littermates (**Figure 2C-D**). To assess the arteriole vessel formation in hindlimbs after HLI, we co-stained gastrocnemius muscle tissue with anti-α-SMA antibody and IB4 to measure the number of α-SMA positive arteriole vessels, as shown in** Figure 2E**. Our investigations showed more arteriole vessels observed in gastrocnemius muscles of the *S1pr2^ECKO^* mice, in comparison with *WT* littermates (**Figure 2E-F**).

Furthermore, histological analysis of gastrocnemius muscles after HLI showed less interstitial fibrosis and more muscle fiber area in *S1pr2^ECKO^* mice compared with *WT* mice, indicating that S1pr2 loss-of-function improved ischemic muscle tissue repair (**Figure 2G-J**). As expected, *S1pr2^ECKO^* mice also improved functional outcomes in their hindlimbs compared with *WT* littermates (Tarlov score 4.25 ± 0.71 in *S1pr2^ECKO^* mice vs. 3.38 ± 0.52 in *WT* mice, P < 0.05 versus *WT* at day 7 after HLI ) and displayed less ischemic damage of the hindlimbs (Ischemic score 4.00 ± 0.76 in *S1pr2^ECKO^* mice vs. 3.13 ± 0.35 in *WT* mice, P < 0.05 versus *WT* mice 14 days after HLI) (**Figure 2K-M**), suggesting that EC-S1pr2 loss-of-function protected the muscle function recovery surrounding the ischemic site. Collectively, these results demonstrated that EC-S1pr2 loss-of-function promoted post-ischemic angiogenesis and blood perfusion recovery, and thus improved tissue repair upon HLI.

### Specific endothelial S1pr2 gain-of-function worsens post-ischemic angiogenesis and reduces blood perfusion

**Figure 3:**
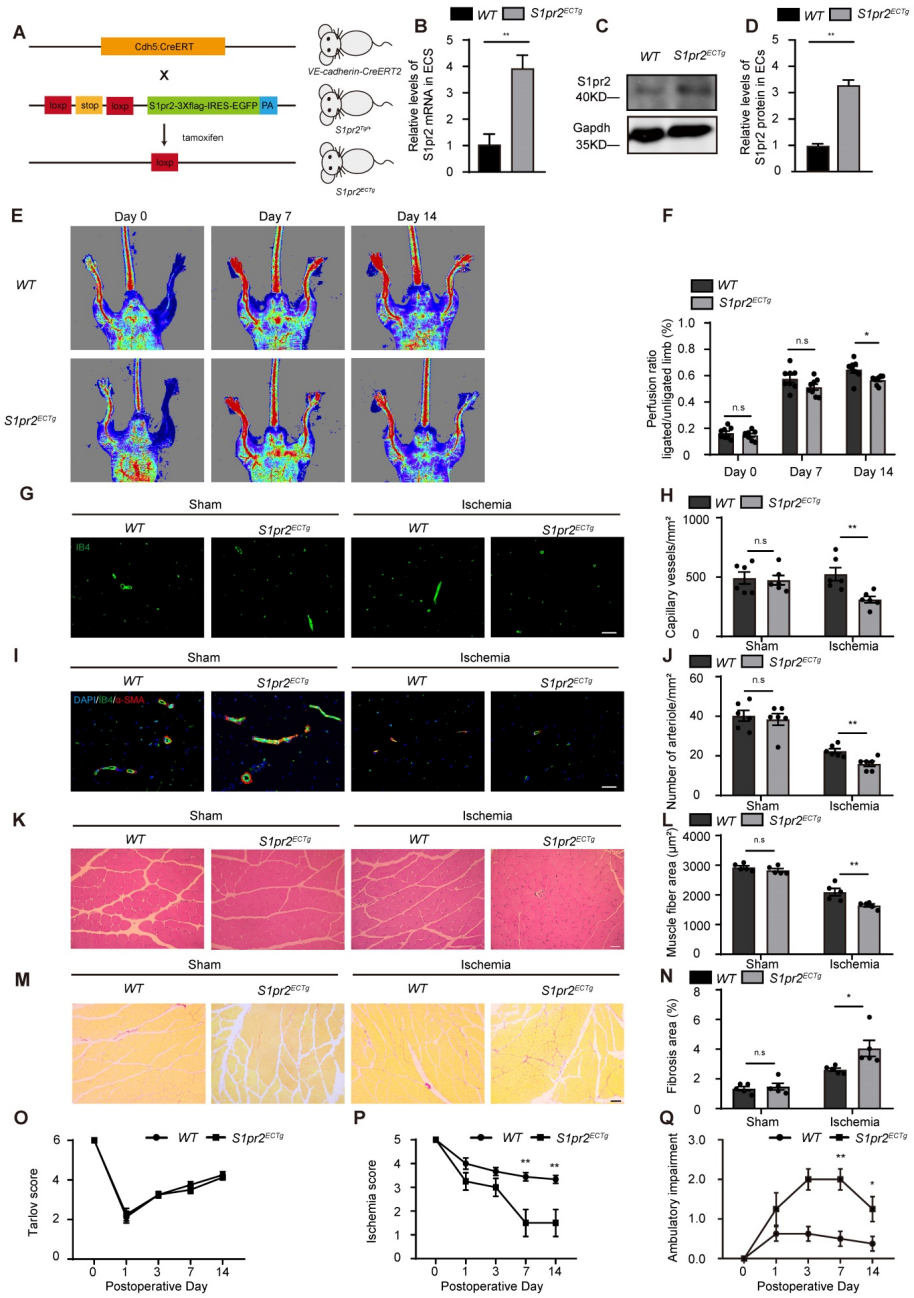
** Specific endothelial S1pr2 gain-of-function reduces post-ischemic angiogenesis and blood flow perfusion after HLI. A,** Generation of EC-specific S1pr2 gain-of-function mice. **B,** Relative mRNA expression levels of S1pr2 in ECs of *WT* and* S1pr2^ECTg^* mice (n = 3).** C-D,** Western blotting analysis of S1pr2 protein levels in ECs from *S1pr2^ECTg^* mice or *WT* control mice, with their quantification (n = 3).** E,** Representative laser Doppler images show impaired blood perfusion in *S1pr2^ECTg^* mice compared with *WT* control mice. **F,** Cumulative results for *WT* mice (n = 8) and *S1pr2^ECTg^* mice (n = 8) are shown as the ratio of blood flow in the ischemic limb to that in the non-ischemic limb at each time point. **G-H,** Representative images of isolectin-B4 staining of gastrocnemius muscles in *WT* and *S1pr2^ECTg^* mice (**G**), with quantification of capillary density in gastrocnemius muscles after sham or HLI operation (**H**) (n = 6).** I-J**, Representative images of α-SMA staining of gastrocnemius muscles in *WT* and *S1pr2^ECTg^* mice (**I**), with quantification of arteriole density in limb muscle after sham or HLI operation (**J**) (n = 6).** K-L,** Representative images of H&E staining of gastrocnemius muscles in *WT* and *S1pr2^ECTg^* mice (**K**), with quantification of muscle fiber area after sham or HLI operation (**L**) (n = 5). **M-N**, Representative images of Sirius red-stained of gastrocnemius muscles in *WT* and *S1pr2^ECTg^* mice (M), with quantification of the percentage of fibrotic tissue in muscle (**N**) (n = 5). **O-Q,** Functional assessment of ischemic muscle over follow-up. Cumulative results for *WT* mice and *S1pr2^ECTg^* mice are shown graphically as Tarlov score (**O**), ischemia score (**P**), and ambulatory impairment score (**Q**) (n = 8). Scale Bars: **G** and **I**, 50 μm; **K** and **M**, 100 μm. Data are mean ± SEM. n.s indicates not significant. *P < 0.05; **P < 0.01.

To further confirm the role of EC-S1pr2 for post-ischemic angiogenesis and tissue repair* in vivo,* we generated EC-specific *S1pr2* gain-of-function mice (*S1pr2^ECTg^*) by crossing* the VE-cadherin-Cre^ERT2^* line with *S1pr2^Tg^* mice (**Figure 3A**). The S1pr2 mRNA levels were sharply increased in ECs of *S1pr2^ECTg^* mice after tamoxifen treatment, as shown by RT-qPCR and western blotting (**Figure 3B-D**). Our *in vivo* data showed that the blood flow perfusion in ischemic hindlimbs of *S1pr2^ECTg^* mice was significantly lower than *WT* littermates (**Figure 3E-F**). Further immunostaining data revealed that capillary density as well as arteriole vessels decreased in the *S1pr2^ECTg^* mice compared with *WT* littermates (**Figure 3G-J**). Together with our results obtained from *S1pr2^ECKO^* mice, these data from* S1pr2^ECTg^* mice confirmed a crucial role of EC-S1pr2 for post-ischemic angiogenesis.

In contrast to EC-S1pr2 loss-of-function, EC-S1pr2 gain-of-function led to a more pronounced interstitial fibrosis and an increased muscle fiber atrophy compared with *WT* control mice (**Figure 3K-N**), indicating that EC-S1pr2 gain-of-function severely impaired post-ischemic muscle tissue repair. Accordingly, *S1pr2^ECTg^* mice had worse ischemic damage of the limb (Ischemia score 1.50 ± 1.60 in *S1pr2^ECTg^* mice vs. 3.44 ± 0.53 in *WT* mice, P < 0.05 versus *WT* mice at day 7 after HLI) and impaired limb function recovery (Ambulatory impairment score 2.00 ± 0.76 in *S1pr2^ECTg^* mice vs. 0.50 ± 0.53 in *WT* mice, P < 0.05 versus *WT* mice at day 7 after HLI) (**Figure 3O-Q**). Collectively, these results demonstrated that EC-S1pr2 gain-of-function hindered post-ischemic angiogenesis and blood perfusion recovery, resulting in severe tissue injury and poor tissue repair after HLI.

### S1pr2 restricts EC migration, proliferation and angiogenic activity

**Figure 4:**
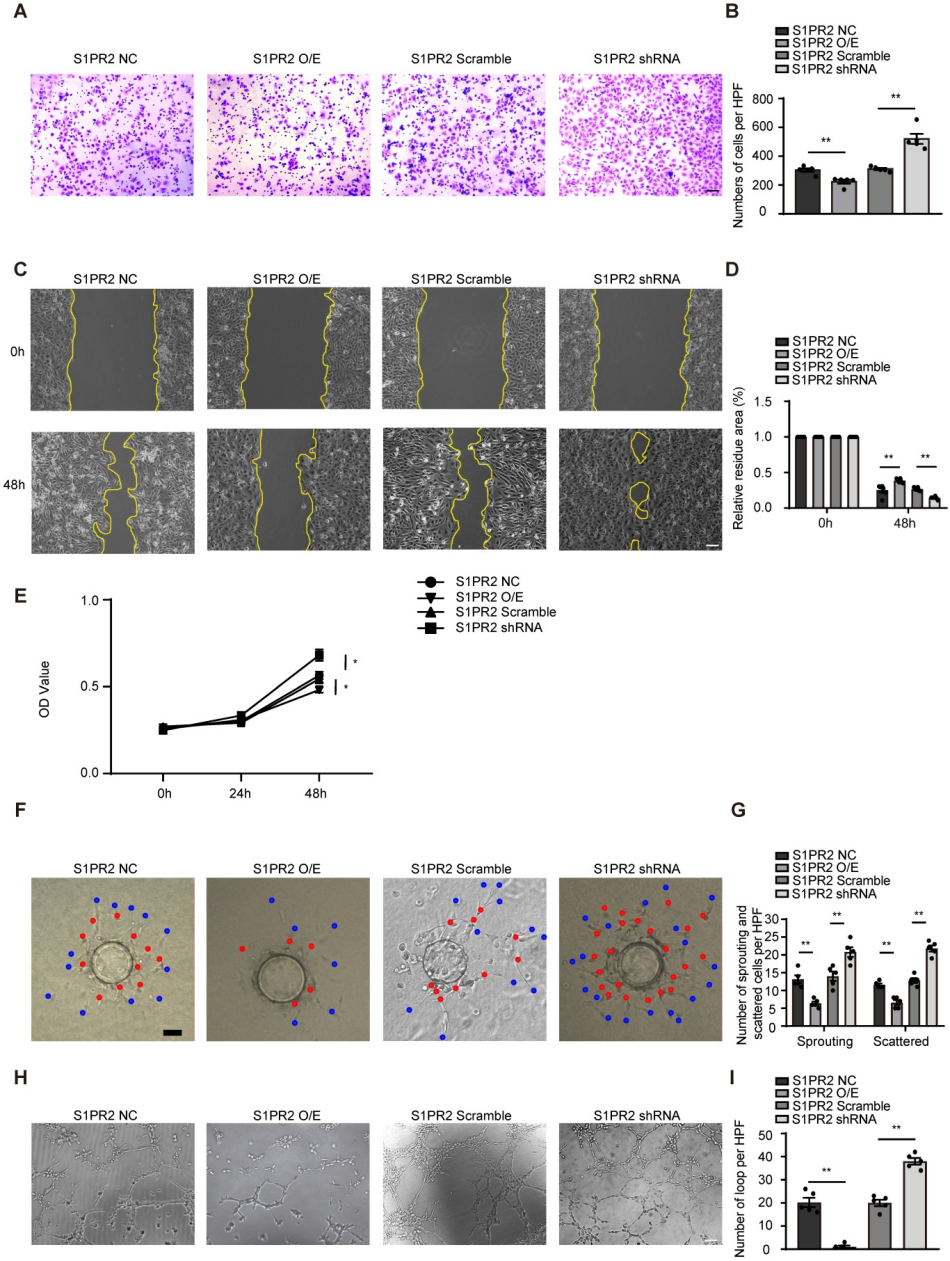
** S1PR2 inhibits EC migration, proliferation, and angiogenic activity. A-D,** S1PR2 inhibits the ability of migration of HUVECs, as shown by transwell chemotactic assay (**A-B**) and scratch wound healing assay (**C-D**) (n = 6). **E**, S1PR2 inhibits the ability of proliferation of HUVECs, as shown by MTT assay (n = 5). **F-G**, S1PR2 inhibits the ability of angiogenic sprouting of HUVECs, as shown by fibrin gel bead sprouting assay (n = 5). **H-I**, S1PR2 inhibits the ability of tube formation of HUVECs, as shown by tube formation assay (n = 5). Scale Bars, 200 μm. Data are mean ± SEM. n.s indicates not significant. *P < 0.05; **P < 0.01.

To dissect molecular mechanisms of EC-S1pr2 expression responsible for post-ischemic angiogenesis in hindlimbs, cell migration/proliferation tests were performed in human umbilical vein endothelial cells (HUVECs). Lentivirus carrying S1PR2 (S1PR2 O/E) or S1PR2 shRNA was generated to achieve elevated or decreased expression of S1PR2 in HUVECs, respectively. Overexpression of S1PR2 in HUVECs markedly decreased cell migration and proliferation, whereas S1PR2 knock-down exerted an enhancing effect on ECs, as shown by the transwell chemotactic assay, the scratch wound healing assay and the MTT assay, respectively (**Figure 4A-E**). Notably, we added 5-FU into all cell migration assays including the transwell chemotactic assay and the scratch wound healing assay to inhibit cell proliferation, suggesting that the inhibitory effect of S1PR2 on EC migration was independent on its effect on EC proliferation. Similar results were obtained from mouse cardiac microvascular endothelial cell line (mCMVECs), which further confirmed that S1pr2 inhibited EC migration and proliferation (**Figure S2A-E**). We next analyzed the effect of S1PR2 on* in vitro* angiogenic activity of ECs by the fibrin gel bead sprouting assay and tube formation assay. Using the fibrin gel bead sprouting assay, we observed markedly attenuated sprout angiogenic activity when S1PR2 was overexpressed in HUVECs, as shown by a significant reduction in sprouts (**Figure 4F-G**, red points) and scattered cells (**Figure 4F-G**, blue points). In contrast to S1PR2 overexpression, enhanced sprouting angiogenesis was observed in HUVEC-expressing S1PR2 shRNA (**Figure 4F-G**). HUVECs with elevated S1PR2 expression resulted in an attenuated angiogenic tube formation, while HUVEC-expressing S1PR2 shRNA increased EC tube formation (**Figure 4H-I**). These results suggest that expression of S1pr2 in ECs restricts cell migration, cell proliferation and angiogenic activity.

### S1pr2 inhibits AKT/eNOS/NO signaling pathway, and thus reduces EC migration, proliferation and angiogenic activity

**Figure 5:**
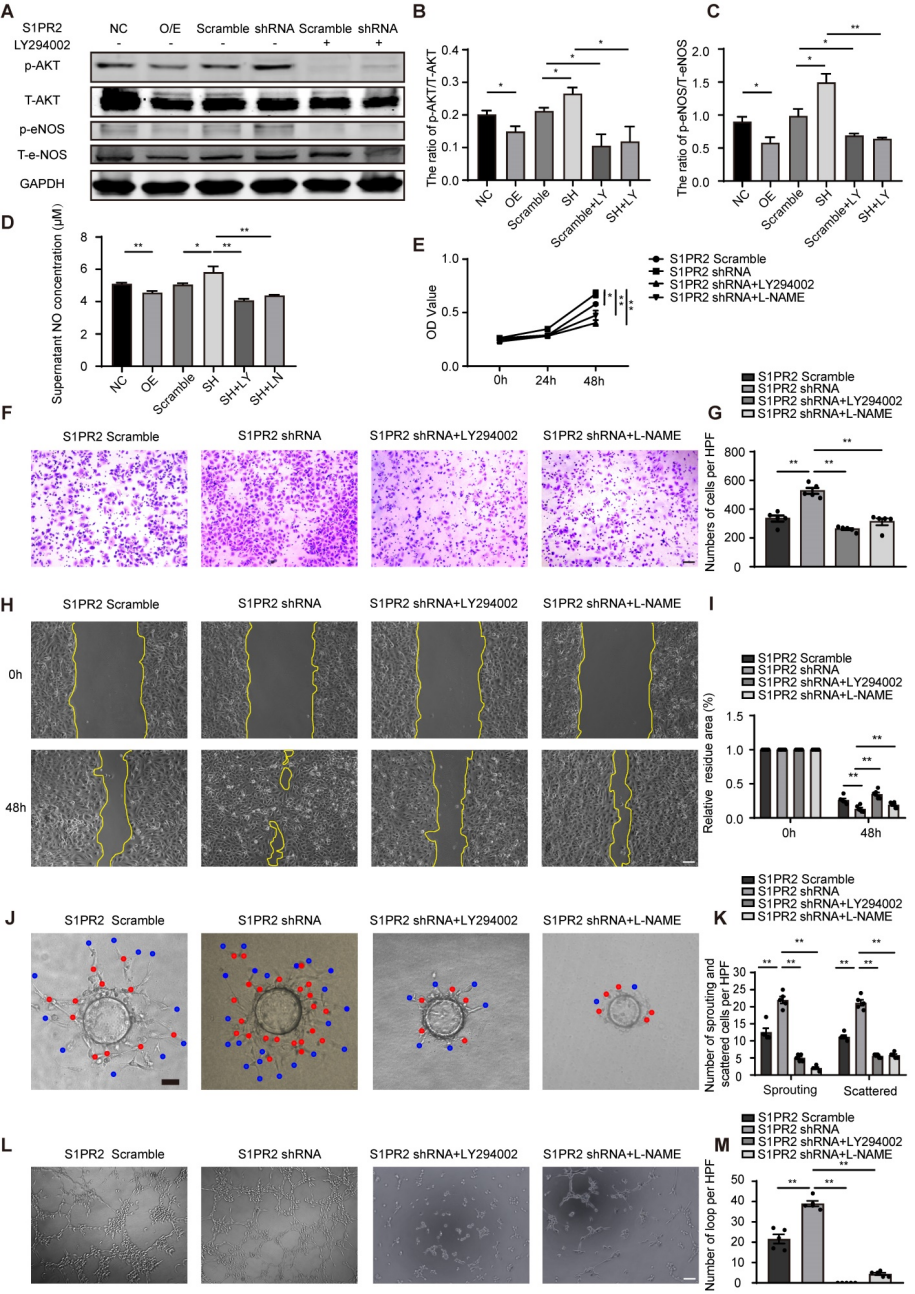
** S1pr2 inhibits AKT/eNOS/NO signaling pathway, and thus reduces EC migration, proliferation, and angiogenic activity. A-C.** Western blotting of AKT or eNOS activation status in HUVECs and quantification in the indicated groups (n = 3). **D.** S1PR2 inhibits the NO production of HUVECs through AKT/eNOS signaling pathway (n = 8). **E.** S1PR2 inhibits the ability of proliferation of HUVECs through AKT/eNOS signaling pathway, as shown by MTT assay (n = 5). F-I, S1PR2 inhibits the ability of migration of HUVECs through AKT/eNOS signaling pathway, as shown by transwell chemotactic assay **(F-G)** and scratch wound healing assay (H-I) (n = 6). **J-K**, S1PR2 inhibits the ability of angiogenic sprouting of HUVECs through AKT/eNOS signaling pathway, as shown by fibrin gel bead sprouting assay (n = 5). **L** and **M**, S1PR2 inhibits the ability of tube formation of HUVECs through AKT/eNOS signaling pathway, as shown by tube formation assay (n = 5). LY294002 (LY), AKT inhibitor. L-NAME (LN), eNOS antagonist. O/E, overexpression. SH, shRNA. Scale Bars, 200 μm. Data are mean ± SEM. n.s indicates not significant. *P < 0.05; **P < 0.01.

It has been shown that S1pr2 was involved in the regulation of AKT signaling pathway 17, 18. To test whether AKT activity might be influenced by S1pr2 in endothelial cells, we performed western blot analysis. Our results showed that the active levels of AKT were significantly lower in S1PR2-overexpressing HUVECs, but higher in S1PR2-silencing HUVECs, demonstrating that S1PR2 down-regulates AKT activation in ECs (**Figure 5A-B**). In endothelial cells, eNOS signaling pathway, as one downstream target of the AKT signaling pathway, contributed to produce NO, which tightly controls endothelial functions 19. We next investigated whether S1PR2 regulated eNOS signaling pathway and NO production in ECs. Our western-blot analysis showed decreased levels of phospho-eNOS in S1PR2-overexpressing HUVECs, but higher levels in S1PR2-silencing HUVECs, confirming that EC-S1PR2 controls eNOS signaling pathway (**Figure 5A-C**). Correspondingly, S1PR2 overexpression inhibited NO production, while S1PR2 knock-down enhanced NO production in ECs (**Figure 5D**) These phenotypes in S1PR2-silencing HUVECs were reversed by AKT inhibitor, LY294002 (**Figure 5A-D**), suggesting that S1PR2-induced down-regulation of eNOS/NO pathway is dependent on AKT signaling pathway. To further investigate whether the AKT/eNOS signaling pathway is required for the effect of EC-S1pr2 on EC migration/proliferation and angiogenic activity, we treated ECs with AKT/eNOS inhibitors in S1PR2-silencing ECs *in vitro* (**Figure 5E-M**). Both AKT inhibitor, LY294002, and eNOS antagonist, L-NAME, reversed the enhanced effect of S1pr2-silencing on EC proliferation/migration, angiogenic sprouting and tube formation *in vitro* (**Figure 5E-M** and **Figure S2C-D**). Taken together, these results indicate that EC-S1pr2 might control post-ischemic angiogenesis by the regulation of NO production via AKT/eNOS signaling pathway.

### Pharmacological inhibition of S1pr2 by JTE013 improves post-ischemic angiogenesis and enhances blood flow perfusion in ischemic tissues

**Figure 6:**
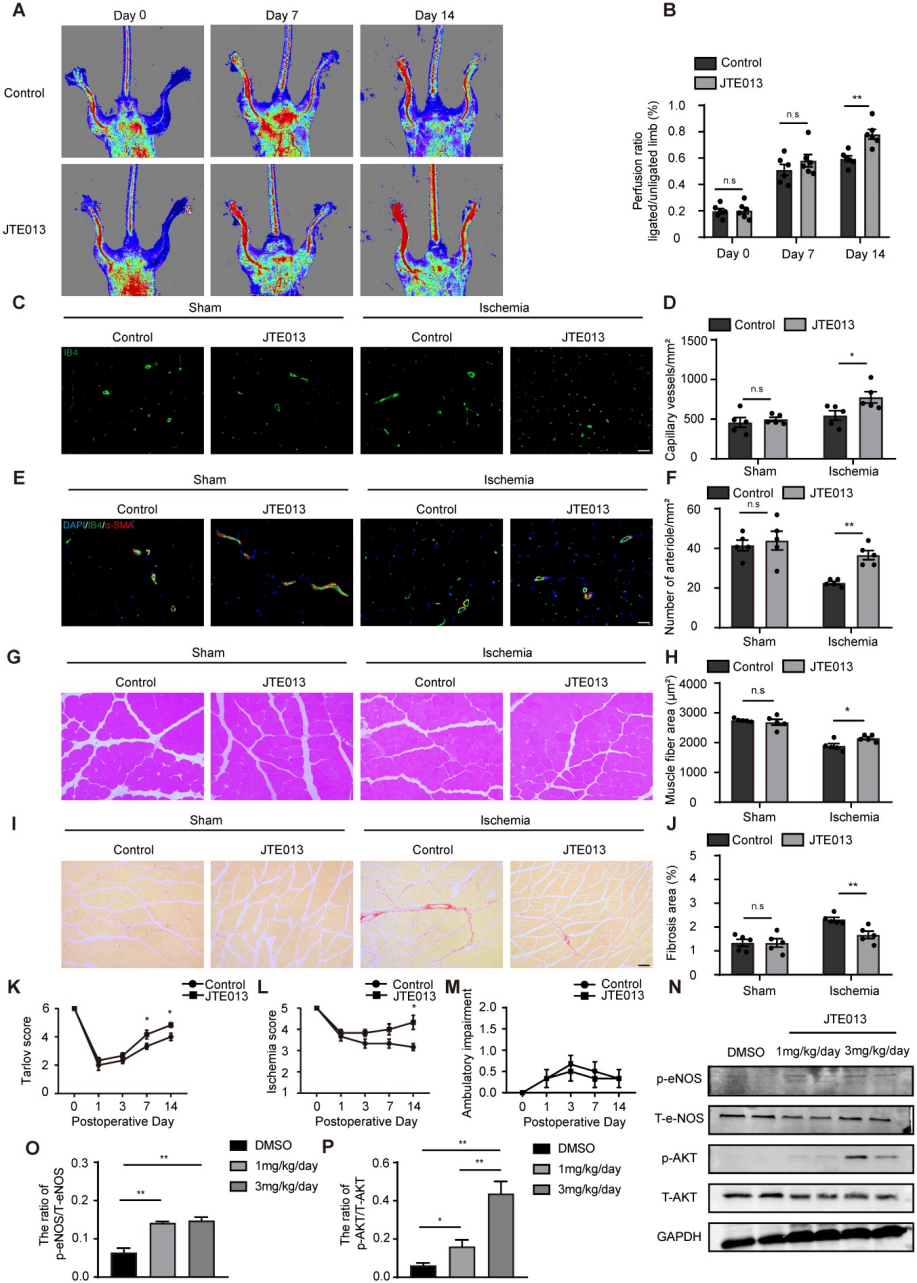
** Pharmacological inhibition of S1pr2 by JTE013 improves post-ischemic angiogenesis and enhances blood flow perfusion in hindlimbs. A**, Representative laser Doppler images show improved blood flow perfusion in JTE013-treated mice compared with control mice. **B,** Cumulative results for control mice (n = 6) and JTE-treated mice (n = 6) are shown graphically as the ratio of blood flow in the ischemic limb to that in the non-ischemic limb at each time point. **C-D,** Representative images of isolectin-B4 staining of gastrocnemius muscles in control mice and JTE013-treated mice, with quantification of capillary density in gastrocnemius muscles after sham or HLI operation (n = 5).** E-F,** Representative images of α-SMA staining of gastrocnemius muscles in control mice and JTE013-treated mice **(E)**, with quantification of arteriole density in gastrocnemius muscles after sham or HLI operation** (F)** (n = 5). **G-H,** Representative images of H&E staining of gastrocnemius muscles in control mice and JTE013-treated mice **(G)**, with quantification of muscle fiber area after sham or HLI operation **(H)** (n = 5). **I-J,** Representative images of Sirius red-stained of gastrocnemius muscles in control mice and JTE013-treated mice **(I)**, with quantification of the percentage of fibrotic tissue in muscle **(J)** (n = 5). K-M, Functional assessment of ischemic muscle over follow-up. Cumulative results for control mice and JTE013-treated mice are shown graphically as Tarlov score **(K)**, ischemia score **(L)**, and ambulatory impairment score **(M)** (n = 6). N-P, Western blotting of AKT or eNOS activation status in hindlimbs of mice treated with JTE013 or DMSO and quantification in the indicated groups (n = 4). Scale Bars: **C** and **E**, 50 μm; **G** and **I**,100 μm. Data are mean ± SEM. n.s indicates not significant. *P < 0.05; **P < 0.01.

Because genetic EC-S1pr2 deficiency can enhance angiogenesis and improve blood flow recovery after ischemia, we next tested whether pharmacological inhibition of S1pr2 had a beneficial effect on hindlimb ischemia. Pharmacological inhibition of S1pr2 by JTE013 for 2 weeks significantly improved blood flow recovery and tissue repair upon HLI, as shown by the laser Doppler perfusion imaging system (**Figure 6A-B**). Our further data showed that the levels of capillary and arteriole vessels significantly increased in mice treated with JTE013, in comparison with control mice (**Figure 6C-F**), suggesting that the pharmacological inhibition of S1pr2 promoted post-ischemic angiogenesis and improves blood perfusion recovery after HLI. Necropsy examination of the gastrocnemius muscles showed that JTE013 resulted in a less interstitial fibrosis and an increased muscle fiber area compared with the control group, demonstrating that inhibition of S1pr2 improves the muscle tissue repair surrounding the ischemic site (**Figure 6G-J**).

As expected, JTE013 improved functional outcomes in their hindlimbs compared with the control group (Tarlov score 4.83 ± 0.41 in the JTE013 group vs. 4.00 ± 0.63 in the control group, P < 0.05 versus the control group at day 14 after HLI) and displayed mild ischemic damage of the limbs (Ischemia score 4.33 ± 0.82 in the JTE013 group vs. 3.17 ± 0.41 in the control group, P < 0.05 versus the control group at day 14 after HLI) (**Figure 6K-M**). Previous investigations and our* in vitro* experiments showed that the inhibition of S1pr2 enhanced the AKT/eNOS pathway activity 18. To further examine the effect of JTE013 on S1pr2 downstream signaling pathway* in vivo*, we detected AKT and eNOS activity in gastrocnemius muscles of these mice treated with JTE013 by western-blotting. Our data showed that JTE013 significantly enhanced both eNOS and AKT activity in a dose-dependent manner* in vivo* (**Figure 6N-P**), suggesting that JTE013 treatment effectively inhibited S1pr2 and affected its downstream targets, which might contribute to its beneficial effects on post-ischemic revascularization. These results demonstrate that the pharmacological inhibition of S1pr2 promotes post-ischemic angiogenesis and blood perfusion recovery, and therefore provides JTE013 as a possible novel therapeutic drug against ischemic diseases.

### RGD-peptide magnetic nanoparticle EC-target delivery of S1pr2-siRNA enhances post-ischemic angiogenesis and blood flow perfusion

**Figure 7:**
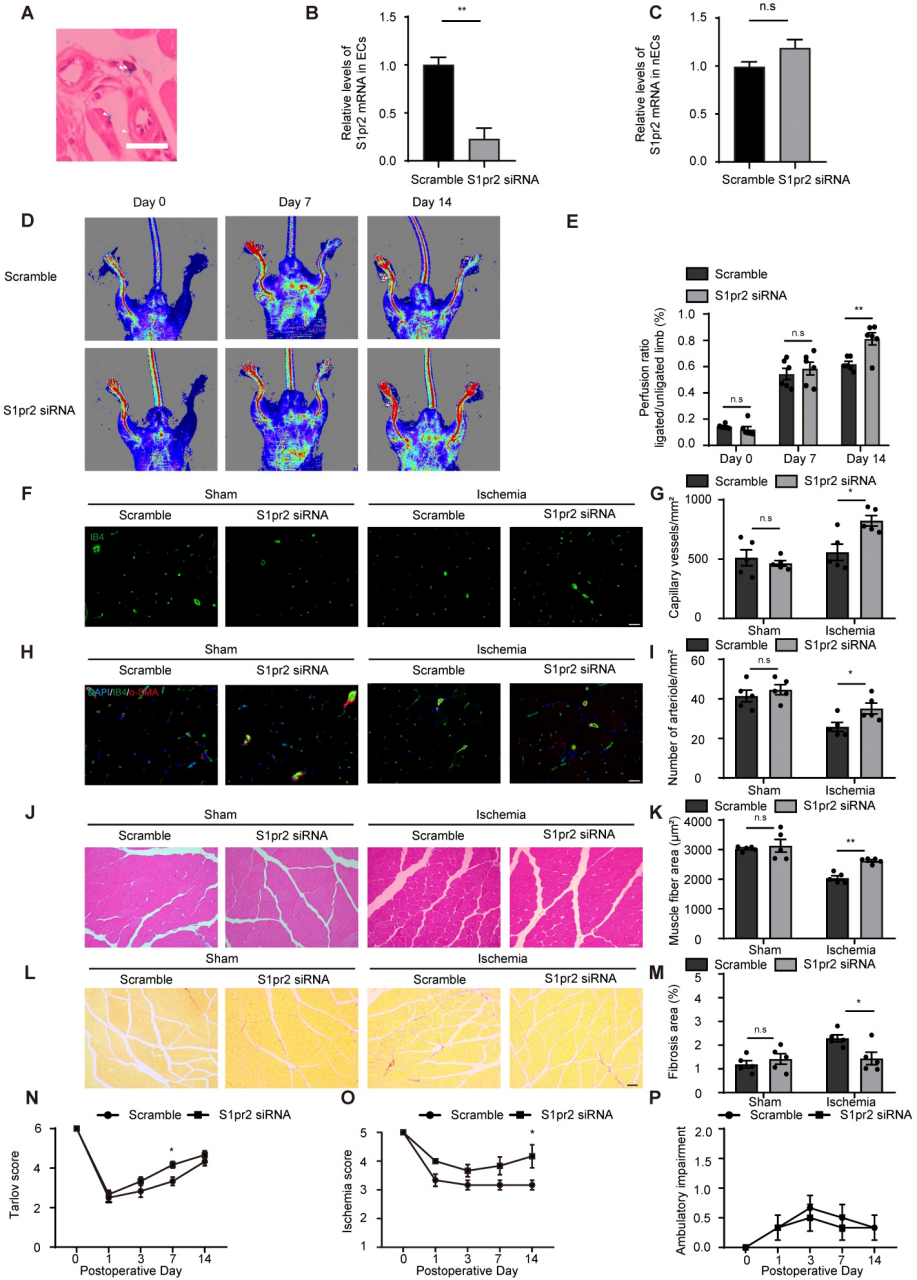
** RGD-peptide magnetic nanoparticle EC target delivery of S1pr2-siRNA enhances post-ischemic angiogenesis and blood flow perfusion in the hindlimb. A,** Perlus staining shown that RGD-Fe_3_O_4_ magnetic nanoparticles (arrowhead) are successfully delivered to hindlimb ECs (n = 3). **B-C,** RGD-nanoparticle packaging S1pr2-siRNA target delivery reduces the expression of S1pr2 **(B),** as shown by RT-qPCR in hindlimb ECs, with no significant change of S1pr2 expression in the remaining hindlimb components when ECs were removed **(C)** (n = 3). **D,** Representative laser Doppler images show improved blood flow perfusion in mice treated with S1pr2-siRNA compared with those treated with scramble-siRNA. **E,** Cumulative results for mice treated with S1pr2-siRNA (n = 6) or scramble-siRNA (n = 6) are shown graphically as the ratio of blood flow in ischemic limb to that in the non-ischemic limb at each time point. **F-G,** Representative images of isolectin-B4 staining of gastrocnemius muscles in mice treated with S1pr2-siRNA or scramble-siRNA **(F)**, with quantification of capillary density in gastrocnemius muscles after sham or HLI operation **(G)** (n = 5). **H-I,** Representative images of α-SMA staining of gastrocnemius muscles in mice treated with S1pr2-siRNA or scramble-siRNA **(H)**, with quantification of arteriole density in gastrocnemius muscles after sham or HLI operation** (I)** (n = 5). **J-K,** Representative images of H&E staining of gastrocnemius muscles in mice treated with S1pr2-siRNA or scramble-siRNA **(J)**, with quantification of muscle fiber area after sham or HLI operation **(K)** (n = 5). **L-M,** Representative images of Sirius red-stained of gastrocnemius muscles in mice treated with S1pr2-siRNA or scramble-siRNA **(L)**, with quantification of the percentage of fibrotic tissue in muscle **(M)** (n = 5). **N-P,** Functional assessment of ischemic muscle over follow-up. Cumulative results for mice treated with S1pr2-siRNA or scramble-siRNA are shown graphically as Tarlov score** (N)**, ischemia score **(O)**, and ambulatory impairment score **(P)** (n = 6). Scale Bars: F and H, 50 μm; J and L,100 μm. Data are mean ± SEM. n.s indicates not significant. *P < 0.05; **P < 0.01.

Since pharmacological inhibition of S1pr2 might cause side effects apart from its enhancing effect on post-ischemic angiogenesis, we designed RGD-peptide magnetic nanoparticles to specifically knockdown S1pr2 in ECs. We have previously reported that RGD-nanoparticles packaging siRNA specifically targeted ECs and displayed sufficient inhibition efficacy on target gene *in vivo* 14. To inhibit S1pr2 function in ECs, we constructed RGD-nanoparticles packaging S1pr2-siRNA. Our results showed that RGD-nanoparticles led to a sharp drop in the expression of S1pr2 levels in ECs, whereas no significant alteration in S1pr2 expression was observed in the remaining cell components of hindlimbs when ECs were removed (**Figure 7A-C**). The 2 mg/kg RGD-nanoparticles were used in this study, and this strategy resulted in a reduction of 76.67% ± 15.91% in the expression of S1pr2 in ECs *in vivo* (**Figure 7A-C**), exerting a high S1pr2 inhibition efficacy of these nanoparticles. Our further results showed that RGD-nanoparticles packaging S1pr2-siRNA significantly improved blood flow recovery (**Figure 7D-E**). Furthermore, histological analysis revealed higher capillary vessel density and denser arteriole vessels in hindlimbs of mice which received S1pr2-siRNA nanoparticles (**Figure 7F-I**). As expected, EC-S1pr2 inhibition by nanoparticles led to a less interstitial fibrosis and an increased muscle fiber area compared with the control group (**Figure 7J-M**). Moreover, EC-target S1pr2 knock-down improved functional outcomes in their hindlimbs compared with the control group (Tarlov score 4.17±0.41 in the S1pr2-siRNA group vs. 3.33 ± 0.52 in the control group, P < 0.05 versus the control group at day 7 after HLI) and displayed mild ischemic damage of the limbs (Ischemic score 4.12 ± 0.98 in the S1pr2-siRNA group vs. 3.17 ± 0.41, P < 0.05 versus the control group at day 14 after HLI) (**Figure 7N-P**). Collectively, this study provides a therapeutic strategy for PAD disease by EC-target delivery of S1pr2-siRNA for inhibition of S1pr2 expression via improving post-ischemic angiogenesis and tissue repair.

## Discussion

It has been well known that S1P tightly regulated endothelial cell functions via S1pr1 6, 7, 20. S1pr1 plays a crucial role in vascular integrity and angiogenesis 6, 7. Loss of EC-S1pr1 results in the enhanced endothelial cell sprouting and formation of ectopic vessel branches, suggesting that EC-S1pr1 acts as a vascular-intrinsic stabilization mechanism during vascular development 6. Our previous study revealed that EC-S1pr1 displayed a similar effect on sprouting angiogenesis in tumor angiogenesis as in developmental angiogenesis 21. In addition to S1pr1, S1pr2 is also expressed in vascular endothelial cells 10. However, the effect of S1pr2 on angiogenesis is not well described.

Our *in vitro* experiments showed that the S1pr2 gain-of-function in ECs inhibited cell migration/proliferation and angiogenic activity, which was well consistent with previous investigation 10, 22, 23.

Previous *in vivo* studies showed that pharmacological inhibition of S1pr2 blocked the development of spontaneous hemorrhagic transformation and protected cerebrovascular integrity in an experimental stroke animal model 24. Using S1pr2 straight knock-out mice, Skoura A *et al*. reported that loss of S1pr2 reduced pathological neovascularization in the vitreous chamber in ischemia-driven retinopathy of the mouse retina 10. The aforementioned investigations indicated that S1pr2 played an important role in EC functions and homeostasis *in vivo* 10, 24, and that S1pr2 might be involved in the regulation of post-ischemic angiogenesis. However, the direct* in vivo* evidence of EC-specific effects of S1pr2 on post-ischemic angiogenesis is still lacking, and whether *in vivo* EC-target modulation of S1pr2 signaling improves post-ischemic blood flow recovery is completely unknown. Herein, we generated both EC-specific S1pr2 loss-of-function and gain-of-function mice. By using these EC-specific transgenic mice, we reported that the deficiency of S1pr2 in ECs led to a significant acceleration of post-ischemic angiogenesis in a HLI model, whereas EC-S1pr2 gain-of-function decreased post-ischemic angiogenesis in mice. Our study provides direct *in vivo* evidence that EC-expressing S1pr2 tightly controls post-ischemic angiogenesis, thereby regulating ischemic tissue repair.

Many studies revealed that endothelial nitric oxide synthase (eNOS), an EC-specific isoform of NO producing enzyme, is a main regulator in EC functions and angiogenesis under both physiological and pathophysiological conditions 25-27. It has been shown that eNOS not only functioned alone to induce EC migration/proliferation but also modulated multiple angiogenic factors, exerting a predominant role in both angiogenesis and vasculogenesis 25. Previous studies showed that S1pr2 inhibited endothelial nitric oxide synthase expression 9, 10. Liu W *et al*. reported that pharmacological inhibition of S1pr2 and small interfering RNA (siRNA) against S1pr2 resulted in enhanced NO levels and eNOS activity 9. In consistence with Liu W *et al*.'s report 9, our study reported that loss-of-function of S1pr2 in ECs significantly enhanced eNOS activity and NO production, whereas S1pr2 gain-of-function in ECs reduced eNOS activation and NO production, providing evidence that EC-expressing S1pr2 regulated eNOS/NO signaling pathway which contributed to the inhibitory effect of S1pr2 on EC functions and angiogenesis. In agreement with these* in vitro* data, we detected a reduction of eNOS activity in gastrocnemius muscle of hindlimbs in mice treated with S1pr2 antagonist after HLI* in vivo*. Previous and our studies demonstrated that the AKT signaling pathway was involved in the activation of eNOS 19, 28, 29. Sanchez T *et al*. previously found that S1pr2 actively regulated the PTEN phosphatase in a Rho GTPase-dependent manner to inhibit cell migration 30. PTEN directly opposes the activity of PI3K by dephosphorylating PIP_3_, which mediates Akt stimulation. Cui H *et al*. reported that S1pr2 negatively regulated the AKT/eNOS pathway and exerted inhibitory effects on NO production in vascular ECs 18. In consistence with these findings, our *in vitro* experiments showed that endothelial S1pr2 inhibited AKT activity and that the inhibition of AKT/eNOS reversed the inhibitory effect of S1pr2 on EC migration/proliferation and angiogenic activity, explaining the molecular mechanism by which EC-S1pr2 restricts post-ischemic angiogenesis through the AKT/eNOS/NO signaling pathway.

S1pr2 antagonist, JTE013, has been reported to exert beneficial effects in various disease models: pharmacological inhibition of S1pr2 retarded the development of spontaneous hemorrhagic transformation and promoted cerebrovascular integrity in an experimental stroke animal model; JTE013 alleviated inflammatory bone loss disease in mice; blockade of S1pr2 attenuated allergic asthma in a mouse model; S1pr2 inhibitor reduced portal vein pressure and protect against liver injury in a rat model 24, 31-33. In addition to these therapeutic effects of the S1pr2 antagonist on different diseases, we found that the S1pr2 inhibitor, JTE013, exerted a protective effect on post-ischemic injury in a HLI mouse model. Administration of JTE013 significantly improved post-ischemic angiogenesis and blood flow perfusion in the PAD model, providing a therapeutic target against S1pr2 as a novel strategy to improve revascularization and blood flow recovery for PAD.

Although pharmacological inhibition of S1pr2 displays beneficial effects on post-ischemic angiogenesis and tissue repair in the HLI mice model, off-target effects of systemic administration of S1pr2 inhibitor might cause potential side effects and toxicity, as observed in other S1p receptor modulators 34. Previous investigations revealed that S1pr2 acted as a tumor suppressor in colorectal cancer 35. Petti L* et al*. reported that JTE013 treatment broadened high-grade adenomas and carcinomas in an intestinal and mammary tumorigenesis model 35, suggesting that systemic inhibition of S1pr2 might result in a potential oncogenesis. To overcome the potential serious off-target adverse effects by systemic administration of S1pr2 chemical antagonist, we developed an EC-target strategy to achieve EC-specific blockade of S1pr2 via RGD-Fe_3_O_4_ nanoparticles. Our experiments presented that RGD-Fe_3_O_4_ nanoparticles successfully delivered S1pr2-siRNA to endothelial cells *in vivo*. RGD-nanoparticles packaging S1pr2-siRNA specifically down-regulated the expression levels of S1pr2 in ECs, not in non-EC tissues. This EC-target inhibition of S1pr2 might not lead to potential side effects caused by systemic administration of S1pr2 chemical antagonist but significantly improve post-ischemic angiogenesis and blood flow reperfusion in a PAD model, therefore providing a promising cell-target therapy for PAD.

**Figure 8:**
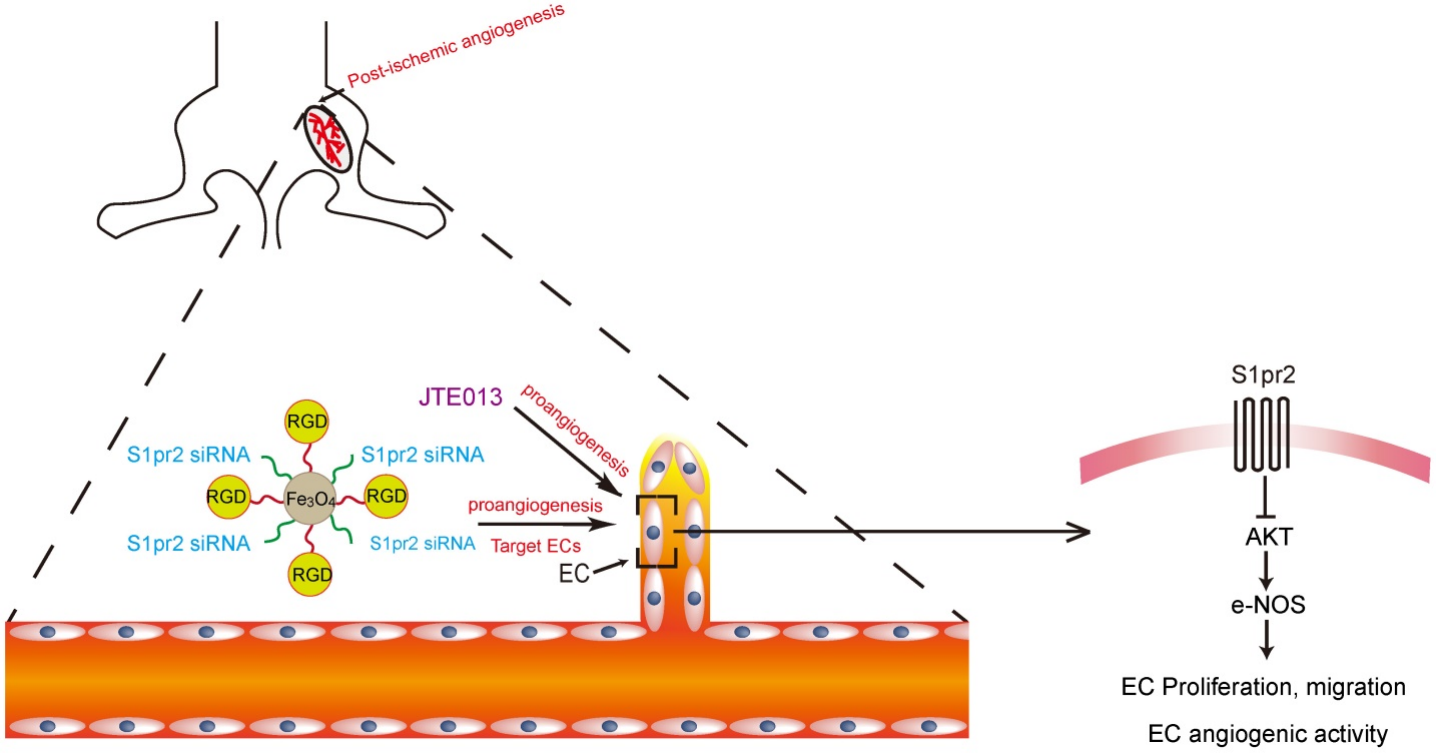
Graphic abstract shows that endothelial cell-expressing S1pr2 plays an important role in post-ischemic angiogenesis and blood flow recovery via AKT/eNOS signaling pathway in peripheral artery disease, and that EC-targeted therapy against S1pr2 might be a potential and novel intervention for patients with peripheral artery disease.

In conclusion, by using both EC-specific S1pr2 loss-of-function and gain-of-function mice, we presented strong *in vivo* evidence to support an essential role of EC-expressing S1pr2 in the regulation of post-ischemic angiogenesis. Mechanically, S1pr2 signaling inhibits AKT/eNOS signaling pathway in ECs, and thus retards EC cell proliferation/migration and angiogenic activity, revealing a novel mechanism through EC-expressing S1pr2 regulates post-ischemic angiogenesis and tissue repair (**Figure 8**). Pharmacological inhibition of S1pr2 by JTE013 significantly improves post-ischemic angiogenesis and blood flow perfusion, providing a novel therapeutic medication against PAD disease. Moreover, we established an EC-target strategy to achieve EC-specific down-regulation of S1pr2 signaling via nanoparticles to overcome potential off-target adverse effects by systemic administration of S1pr2 antagonist. This EC-target knockdown of S1pr2 treatment significantly improved post-ischemic angiogenesis and blood flow reperfusion, thereby providing a novel cell-target strategy for patients with peripheral artery disease.

## Supplementary Material

Supplementary figures.

## Figures and Tables

**Table 1:** Tarlov Score

Tarlov Score	
0	No movement
1	Barely perceptible movement, non-weight bearing
2	Frequent movement, non-weight bearing
3	Supports weight, partial weight bearing
4	Walks with a mild deficit
5	Normal but slow walking
6	Full and fast walking

**Table 2:** Ischemia Score

Ischemia Score	
0	Auto-amputation > half lower limb
1	Gangrenous tissue > half foot
2	Gangrenous tissue < half foot, with lower limb muscle necrosis
3	Gangrenous tissue < half foot, without lower limb muscle necrosis
4	Pale foot or gait abnormalities
5	Normal

**Table 3:** Ambulatory Impairment Score

Ambulatory Impairment Score	
0	Flexing the toes to resist gentle traction on the tail
1	Plantar flexion
2	No dragging but no plantar flexion
3	Dragging of foot

**Table 4:** Primer list

hS1PR2 F	CATCCTCCTTCTGGACTATGC
hS1PR2 R	GTGTAGATGACGGGGTTGAG
hGAPDH F	GGAGCGAGATCCCTCCAAAAT
hGAPDH R	GGCTGTTGTCATACTTCTCATGG
mS1pr1F	ATGGTGTCCACTAGCATCCC
mS1pr1R	CGATGTTCAACTTGCCTGTGTAG
mS1pr2 F	TGTTGCTGGTCCTCAGACGCTAG
mS1pr2 R	CCAGAAATGTCGGTGATGTAGGC
mS1pr3F	ACTCTCCGGGAACATTACGAT
mS1pr3R	CCAAGACGATGAAGCTACAGG
mS1pr4F	CTGGCTACTGGCAGCTATCC
mS1pr4R	AGACCACCACACAAAAGAGCA
mS1pr5F	TGGCTAACTCGCTGCTGAATC
mS1pr5R	TCGCTGCAAGCTGTTGGAG
mGapdh F	AAATGGTGAAGGTCGGTGTGAACG
mGapdh R	ATCTCCACTTTGCCACTGC
