# Supplementary material for: Endothelial S1pr2 regulates post-ischemic angiogenesis via AKT/eNOS signaling pathway
Source: Theranostics. 2022 Jul 4;12(11):5172–88. doi: 10.7150/thno.71585 (PMC9274736; doi:10.7150/thno.71585)
Supplement: Supplementary file 1 — Supplementary figures. [file thnov12p5172s1.pdf]

Supplementary Figure 1

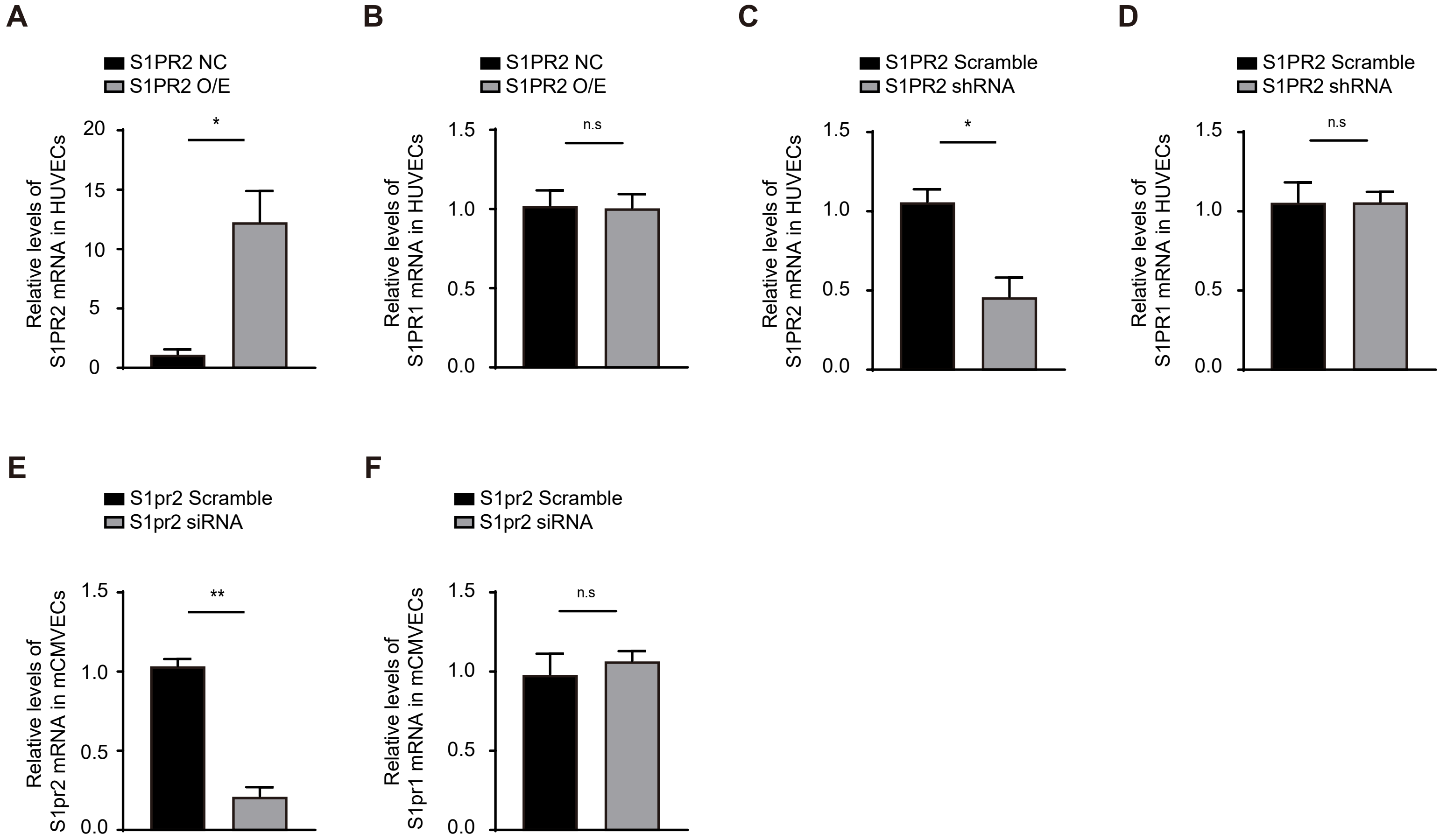

Supplementary Figure 1. The efficiency of S1PR2 knockdown and overexpression in ECs transfected with vectors carrying S1PR2/S1pr2 or their shRNA/siRNA. A-D, Relative mRNA expression levels of S1PR2 and S1PR1 in HUVECs infected with S1PR2 over-expressing (O/E) lentivirus and its corresponding NC empty control lentivirus (**A-B**), and S1PR2 shRNA lentivirus and its corresponding scramble control lentivirus (**C-D**) (n = 3). **E-F,** Relative mRNA expression levels of S1pr2 and S1pr1 in mCMVECs transfected with S1pr2 siRNA or its corresponding scramble siRNA (n = 3). Data are mean ± SEM. n.s indicates not significant. *P < 0.05; **P < 0.01.

Supplementary Figure 2

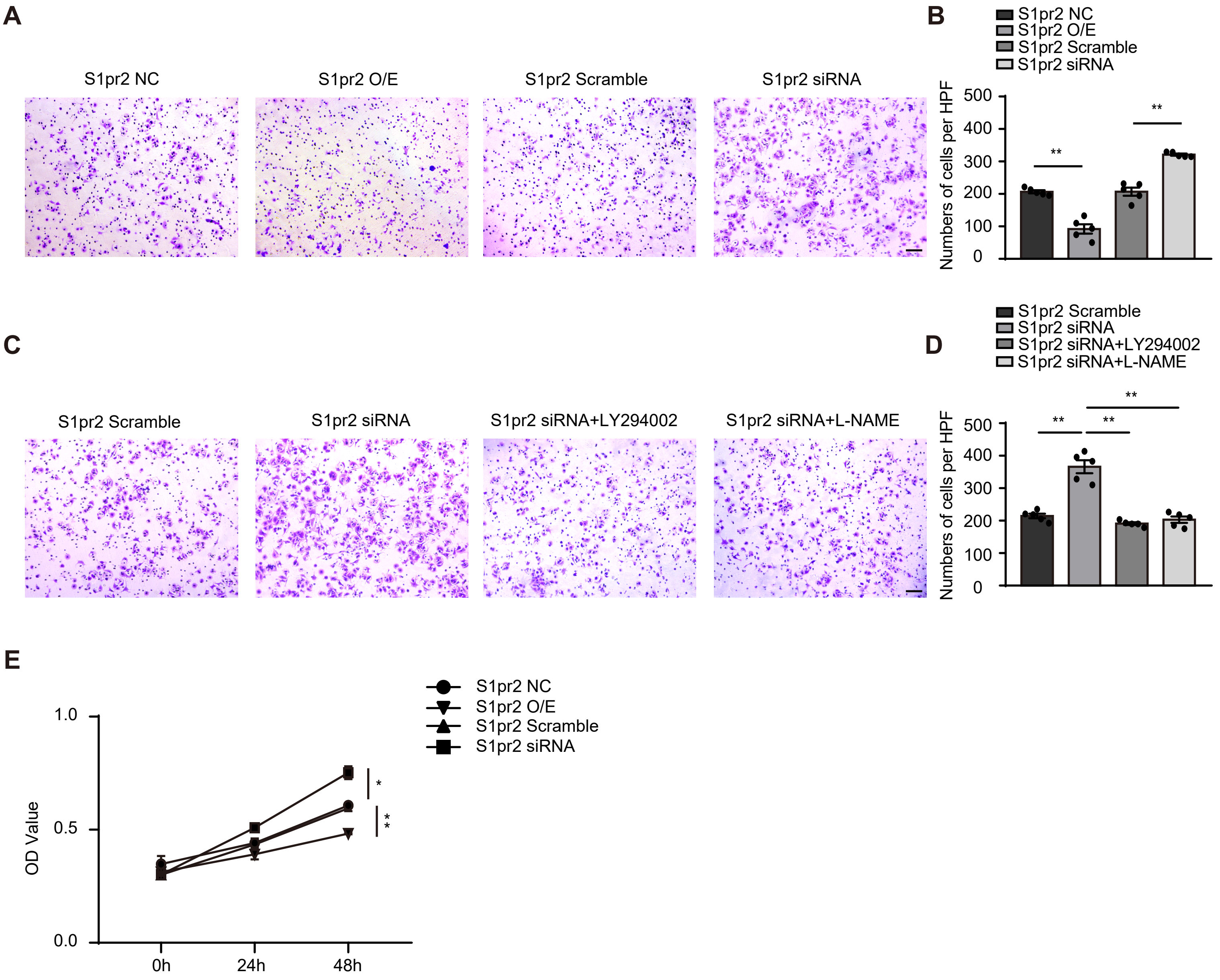

Supplementary Figure 2. S1pr2 inhibits cell migration and proliferation in mouse cardiac microvascular endothelial cell line (mCMVECs). A-B, S1pr2 inhibits the ability of cell migration in mCMVECs, as shown by transwell chemotactic assay (n = 5). **C-D,** S1pr2 inhibits the ability of migration of mCMVECs through AKT/eNOS signaling pathway, as shown by transwell chemotactic assay (n = 5). LY294002, AKT inhibitor. L-NAME, eNOS antagonist. **E**, S1pr2 inhibits the ability of cell proliferation of mCMVECs, as shown by MTT assay (n = 5). Scale Bars, 200 μm. Data are mean ± SEM. n.s indicates not significant. *P < 0.05; **P < 0.01.
